# Dissecting estimation of conductances in subthreshold regimes

**DOI:** 10.1186/1471-2202-15-S1-P151

**Published:** 2014-07-21

**Authors:** Catalina Vich, Antoni Guillamon

**Affiliations:** 1 Dept. of Mathematics and Computer Science, Universitat de les Illes Balears, Palma, 07122, Spain; 2 Dept. of Applied Mathematics I, EPSEB, Universitat Politècnica de Catalunya, 08028, Barcelona, Spain

## 

**Figure 1:**
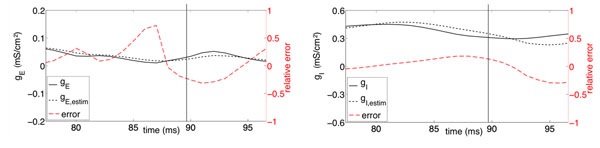
Representation of the relative errors in the I_AHP_ and I_LTS_ dominance phases. The pair of figures shows the relative error (dashed red), the estimated value (dotted black) and the actual value (solid black) of the synaptic, excitatory and inhibitory conductances, respectively, in the subthreshold regime. Vertical lines show the border between the I_AHP_-dominance (left) and I_LTS_-dominance (right) phases. The curves have been smoothed for a clearer plot. The actual values can be found in Table 1.

We study the influence of subthreshold activity in the estimation of synaptic conductances when linear regression methods based on the current-voltage relationship are used. It is known that differences between actual conductances and the estimated ones using such methods can be huge in spiking regimes, so caution has been taken to remove spiking activity from many experimental data before proceeding to linear estimation. However, not much attention has been paid to the influence of ionic currents active in the non-spiking regime. We use a conductance-based model endowed both with an afterhyperpolarizing current and a low subthreshold current to show that the activity of these currents during subthreshold activity can lead to significant errors in synaptic conductance estimation (see Table 1 and Figure 1). More precisely, we found errors higher than 100% in the estimation of excitatory conductances and up to 30% approximately for inhibitory conductances, mean errors being also significantly large. Therefore, conclusions obtained from experimental data by means of this estimation procedure need to be properly reanalyzed.

**Table 1:** Statistics of relative errors in the estimation of total, excitatory and inhibitory conductances.

		I_AHP_ – dominated	I_LTS_ – dominated
Mean / Std deviation	g_syn_	8.6% / 6.45%	-11.23% / 9.69%
	
	g_E_	27.82% / 35.14%	-2.85% /30.48%
	
	g_I_	10.12% /9.07%	-13.87% / 16.30%

Maximum relative error	g_syn_	21.18%	-22.59%
	
	g_E_	109.07%	63.67%
	
	g_I_	24.75%	-32.34%

Our results add a new warning message when extracting conductance traces from intracellular recordings and the conclusions concerning neuronal activity that can be drawn from them. It also stimulates challenging questions in developing theoretical efficient methods to properly estimate synaptic conductances from voltage traces.

